# County-Level COVID-19 Vaccination Coverage and Social Vulnerability — United States, December 14, 2020–March 1, 2021

**DOI:** 10.15585/mmwr.mm7012e1

**Published:** 2021-03-26

**Authors:** Michelle M. Hughes, Alice Wang, Marissa K. Grossman, Eugene Pun, Ari Whiteman, Li Deng, Elaine Hallisey, J. Danielle Sharpe, Emily N. Ussery, Shannon Stokley, Trieste Musial, Daniel L. Weller, Bhavini Patel Murthy, Laura Reynolds, Lynn Gibbs-Scharf, LaTreace Harris, Matt D. Ritchey, Robin L. Toblin

**Affiliations:** ^1^CDC COVID-19 Response Team; ^2^Geospatial Research, Analysis, and Services Program, Agency for Toxic Substances and Disease Registry, Atlanta, Georgia; ^3^General Dynamics Information Technology, Falls Church, Virginia.

The U.S. COVID-19 vaccination program began in December 2020, and ensuring equitable COVID-19 vaccine access remains a national priority.* COVID-19 has disproportionately affected racial/ethnic minority groups and those who are economically and socially disadvantaged (*1*,*2*). Thus, achieving not just vaccine equality (i.e., similar allocation of vaccine supply proportional to its population across jurisdictions) but equity (i.e., preferential access and administra­tion to those who have been most affected by COVID-19 disease) is an important goal. The CDC social vulnerability index (SVI) uses 15 indicators grouped into four themes that comprise an overall SVI measure, resulting in 20 metrics, each of which has national and state-specific county rankings. The 20 metric-specific rankings were each divided into lowest to highest tertiles to categorize counties as low, moderate, or high social vulnerability counties. These tertiles were combined with vaccine administration data for 49,264,338 U.S. residents in 49 states and the District of Columbia (DC) who received at least one COVID-19 vaccine dose during December 14, 2020–March 1, 2021. Nationally, for the overall SVI measure, vaccination coverage was higher (15.8%) in low social vulnerability counties than in high social vulnerability counties (13.9%), with the largest coverage disparity in the socioeconomic status theme (2.5 percentage points higher coverage in low than in high vulnerability counties). Wide state variations in equity across SVI metrics were found. Whereas in the majority of states, vaccination coverage was higher in low vulnerability counties, some states had equitable coverage at the county level. CDC, state, and local jurisdictions should continue to monitor vaccination coverage by SVI metrics to focus public health interventions to achieve equitable coverage with COVID-19 vaccine.

COVID-19 vaccine administration data are reported to CDC by multiple entities via immunization information systems (IIS), the Vaccine Administration Management System, or direct data submission.^†^ Vaccination coverage was defined as the number of residents who received at least one dose of COVID-19 vaccine during December 14, 2020–March 1, 2021, and whose data were reported to CDC by March 6, 2021.^§^ Total county population denominators used to create vaccination coverage estimates were obtained from the U.S. Census Bureau 2019 Population Estimates Program.^¶^ Social vulnerability data were obtained from the CDC SVI 2018 database,** which includes metrics to identify communities that might need additional support during emergencies, including the COVID-19 pandemic (Supplementary Figure 1, https://stacks.cdc.gov/view/cdc/104111). County-level social vulnerability rankings for 15 SVI indicators, four SVI themes, and the overall SVI (20 total SVI metrics) were used.^††^ Each of the SVI metrics was categorized into national^§§^ and state-specific^¶¶^ tertiles*** (low, moderate, and high social vulnerability) based on their national (among all U.S. counties) or state (among each state’s counties) rank.

Vaccination coverage (percentage of residents who received at least one COVID-19 vaccine dose) and 95% confidence intervals (CIs) within SVI tertiles were calculated for each of the 20 SVI metrics for the national analyses, with jurisdictional exclusions based on missing data for state of residence, missing data for county of residence (Hawaii, which did not systematically report these data), or no available SVI metrics (eight territories and freely associated states).^†††^ A vaccination rate ratio (RR) and 95% CI for each SVI metric was calculated using Wald’s unconditional maximum likelihood estimation to assess the relative differences in vaccination coverage, comparing low and moderate vulnerability counties with high vulnerability counties. The rate difference was also calculated to assess the difference between SVI tertiles. Because of the large sample sizes, rather than using statistical significance to determine meaningful differences between tertiles, a difference of ≥0.5 percentage points was used. State-level analyses for the overall SVI and four SVI themes were conducted among states with more than three counties. In addition, vaccination coverage for SVI metrics (national analyses) and SVI metrics within states (state-level analyses) were normalized so that the sum across tertiles was one.^§§§^ (When vaccination coverage is equally distributed among tertiles within an SVI metric, the proportion of persons vaccinated in each SVI tertile is 0.33.) This activity was reviewed by CDC and was conducted consistent with applicable federal law and CDC policy.^¶¶¶^

During December 14, 2020–March 1, 2021, a total of 51,873,700 residents of 49 U.S. states and DC received at least one dose of COVID-19 vaccine. County of residence was available for 95.0% (49,264,338) of these records for analysis. National first-dose vaccination coverage was 15.1%. For overall SVI, vaccination coverage was 1.9 percentage points higher in low vulnerability counties than in high vulnerability counties (15.8% versus 13.9%, respectively) (Table). The same pattern was found for the SVI themes of socioeconomic status, household composition and disability status, and racial/ethnic minority status and language, with the largest vaccination coverage disparity in the socioeconomic status theme (difference of 2.5 percentage points). Vaccination coverage was ≥0.5 percentage points lower in low vulnerability counties than in high vulnerability counties for the following indicators: 1) population aged ≥65 years (2.3 percentage points lower), 2) multiunit housing (1.3 percentage points lower), and 3) households with no vehicle (0.7 percentage points lower) (Figure 1). Indicators associated with similar coverage in low and high vulnerability counties were 1) percentage of persons with a disability and 2) percentage of persons who speak English “less than well.” Vaccination coverage was higher in low vulnerability counties than in high vulnerability counties for the remaining 10 indicators. Among socioeconomic status indicators, the largest disparity was the percentage of adults without a high school diploma (difference of 2.8 percentage points between high and low vulnerability counties). The majority of vaccination coverage differences between tertiles were <2 percentage points.

**TABLE:** Association between county-level COVID-19 vaccination coverage and social vulnerability index (SVI) metrics among persons who received at least one vaccine dose (N = 49,264,338) — United States, December 14, 2020–March 1, 2021*

SVI metric^†^	Vaccination coverage estimate^§^ (95% CI)			Rate ratio for relative differences in vaccination coverage (95% CI)**		Rate differences in vaccination coverage^††^	
	Low social vulnerability^¶^	Moderate social vulnerability^¶^	High social vulnerability^¶^	Low versus high estimate	Moderate versus high estimate	Low–high	Moderate–high
**Overall SVI**	**15.8 (15.83−15.84)**	**15.6 (15.57−15.59)**	**13.9 (13.89−13.90)**	**1.1 (1.14−1.14)**	**1.1 (1.12−1.12)**	**1.94**	**1.69**
**Socioeconomic status**							
**Total**	**15.9 (15.91−15.92)**	**15.0 (14.97−14.98)**	**13.5 (13.45−13.46)**	**1.2 (1.18−1.18)**	**1.1 (1.11−1.11)**	**2.46**	**1.52**
Poverty	15.9 (15.85−15.86)	14.8 (14.79−14.80)	14.2 (14.21−14.23)	1.1 (1.11−1.12)	1.0 (1.04−1.04)	1.64	0.58
Unemployment	15.4 (15.38−15.40)	15.3 (15.30−15.31)	14.5 (14.54−14.55)	1.1 (1.06−1.06)	1.1 (1.05−1.05)	0.85	0.76
Per capita income	15.6 (15.57−15.58)	14.4 (14.35−14.37)	13.5 (13.45−13.48)	1.2 (1.16−1.16)	1.1 (1.07−1.07)	2.11	0.90
No high school diploma	16.0 (16.01−16.02)	15.3 (15.26−15.27)	13.2 (13.22−13.23)	1.2 (1.21−1.21)	1.2 (1.15−1.16)	2.79	2.04
**Household composition and disability status**							
**Total**	**15.6 (15.62−15.63)**	**14.4 (14.41−14.42)**	**14.2 (14.20−14.22)**	**1.1 (1.10−1.10)**	**1.0 (1.01−1.02)**	**1.42**	**0.21**
Age ≥65 yrs	14.6 (14.58−14.59)	15.9 (15.89−15.91)	16.9 (16.90−16.92)	0.9 (0.86−0.86)	0.9 (0.94−0.94)	−2.32	−1.01
Age ≤17 yrs	16.6 (16.57−16.58)	15.5 (15.51−15.53)	13.6 (13.56−13.57)	1.2 (1.22−1.22)	1.1 (1.14−1.14)	3.01	1.95
Disability	15.1 (15.13−15.14)	15.0 (14.95−14.97)	14.9 (14.88−14.90)	1.0 (1.02−1.02)	1.0 (1.00−1.01)	0.24	0.07
Single parent	16.7 (16.68−16.70)	15.6 (15.55−15.56)	14.0 (13.99−14.00)	1.2 (1.19−1.19)	1.1 (1.11−1.11)	2.70	1.56
**Racial/Ethnic minority status and language**							
**Total**	**15.5 (15.45−15.48)**	**15.6 (15.56−15.58)**	**14.9 (14.90−14.91)**	**1.0 (1.04−1.04)**	**1.0 (1.04−1.05)**	**0.57**	**0.67**
Racial/Ethnic minority	15.5 (15.51−15.54)	15.7 (15.66−15.67)	14.8 (14.75−14.76)	1.1 (1.05−1.05)	1.1 (1.06−1.06)	0.77	0.91
Limited English	15.3 (15.30−15.33)	15.5 (15.47−15.49)	14.9 (14.93−14.93)	1.0 (1.02−1.03)	1.0 (1.04−1.04)	0.38	0.55
**Housing type and transportation**							
**Total**	**14.8 (14.81−14.82)**	**15.3 (15.25−15.26)**	**15.0 (15.03−15.05)**	**1.0 (0.98−0.99)**	**1.0 (1.01−1.01)**	**−0.23**	**0.21**
Multiunit housing	14.0 (13.96−13.99)	14.5 (14.49−14.51)	15.2 (15.24−15.24)	0.9 (0.92−0.92)	1.0 (0.95−0.95)	−1.26	−0.74
Mobile homes	15.2 (15.22−15.23)	15.1 (15.05−15.07)	14.0 (13.98−14.00)	1.1 (1.09−1.09)	1.1 (1.08−1.08)	1.24	1.07
Crowding	16.1 (16.08−16.10)	15.1 (15.09−15.11)	14.7 (14.65−14.66)	1.1 (1.10−1.10)	1.0 (1.03−1.03)	1.43	0.45
No vehicle	14.5 (14.49−14.51)	15.4 (15.35−15.36)	15.2 (15.15−15.16)	1.0 (0.96−0.96)	1.0 (1.01−1.01)	−0.66	0.20
Group quarters	15.9 (15.85−15.86)	14.8 (14.79−14.80)	14.2 (14.21−14.23)	1.1 (1.11−1.12)	1.0 (1.04−1.04)	1.64	0.58

**Abbreviation:** CI = confidence interval.

* Vaccines administered to residents of 49 U.S. states (excluding Hawaii) and the District of Columbia during December 14, 2020–March 1, 2021, and reported to CDC by March 6, 2021.

^†^ SVI ranks counties according to 15 social factors (indicators): 1) percentage of persons with incomes below poverty threshold, 2) percentage of civilian population (aged ≥16 years) that is unemployed, 3) per capita income, 4) percentage of persons aged ≥25 years with no high school diploma, 5) percentage of persons aged ≥65 years, 6) percentage of persons aged ≤17 years, 7) percentage of civilian noninstitutionalized population with a disability, 8) percentage of single-parent households with children aged <18 years, 9) percentage of persons who are racial/ethnic minorities (all persons except non-Hispanic White), 10) percentage of persons aged ≥5 years who speak English “less than well,” 11) percentage of housing in structures with ≥10 units (multiunit housing), 12) percentage of housing structures that are mobile homes, 13) percentage households with more persons than rooms (crowding), 14) percentage of households with no vehicle available, and 15) percentage of persons in group quarters. Estimates are created using 2014–2018 (5-year) data from the American Community Survey. The 15 indicators are categorized into four themes: 1) socioeconomic status (indicators 1–4), 2) household composition and disability (indicators 5–8), 3) racial/ethnic minority status and language (indicators 9 and 10), and 4) housing type and transportation (indicators 11–15). Overall SVI includes all 15 indicators as a composite measure. Additional details are available (https://www.atsdr.cdc.gov/placeandhealth/svi/documentation/SVI_documentation_2018.html).

^§^ Total county population denominators used to create vaccination coverage estimates were obtained from the U.S. Census Bureau 2019 Population Estimates Program (https://www.census.gov/data/datasets/time-series/demo/popest/2010s-counties-total.html). Vaccination coverage was calculated as the total number of vaccine doses administered divided by the total population size for included counties in each SVI tertile.

^¶^ Counties were assigned to tertiles (low, moderate, and high social vulnerability) for each of the 20 SVI ranking metrics.

** Rate ratios compare the relative difference in vaccination coverage between SVI tertiles; high social vulnerability is the reference category.

^††^ Rate differences compare the difference in vaccination coverage between SVI tertiles; high social vulnerability is the reference category. Vaccination coverage differences of ≥0.5 percentage points were considered meaningful differences between SVI tertiles.

**FIGURE 1:**
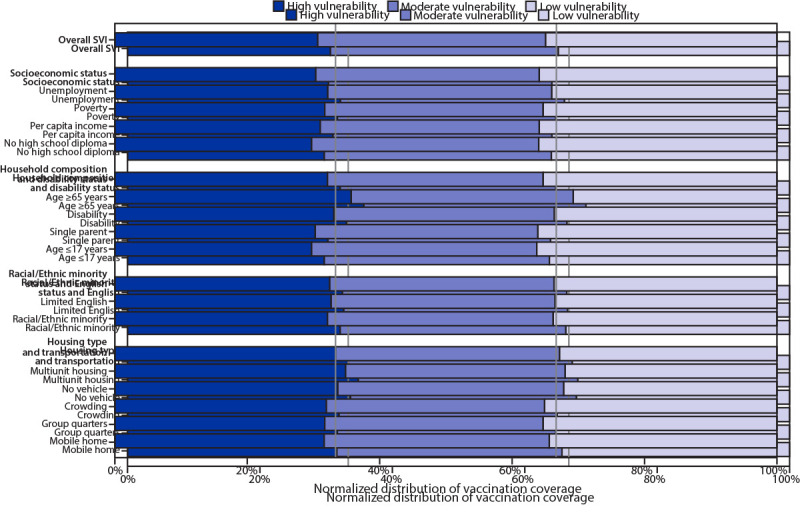
Distribution of county-level* COVID-19 vaccination coverage among persons who received at least one vaccine dose (N = 49,264,338),^†^ by social vulnerability index (SVI) metric^§^ and tertile — United States, December 14, 2020–March 1, 2021 * Counties were assigned to tertiles (low, moderate, and high) for overall SVI. Data are presented as a 100% stacked bar chart (normalized across states), with the length of each bar segment representing the proportion of total vaccination coverage for each SVI tertile. When proportions of vaccination coverage are equal among SVI tertiles, each proportion represents 0.33, represented by the vertical lines. When proportions of vaccination coverage estimates are not equally distributed among SVI tertiles, then proportions do not align with threshold lines representing 0.33. ^†^ Vaccines administered to residents of 49 U.S. states (excluding Hawaii) and the District of Columbia during December 14, 2020–March 1, 2021, and reported to CDC by March 6, 2021. ^§^ SVI ranks counties according to 15 social factors (indicators): 1) percentage of persons with incomes below poverty threshold, 2) percentage of civilian population (aged ≥16 years) that is unemployed, 3) per capita income, 4) percentage of persons aged ≥25 years with no high school diploma, 5) percentage of persons aged ≥65 years, 6) percentage of persons aged ≤17 years, 7) percentage of civilian noninstitutionalized population with a disability, 8) percentage of single-parent households with children aged <18 years, 9) percentage of persons who are racial/ethnic minorities (i.e., all persons except those who are non-Hispanic White), 10) percentage of persons aged ≥5 years who speak English “less than well,” 11) percentage of housing in structures with ≥10 units (multiunit housing), 12) percentage of housing structures that are mobile homes, 13) percentage households with more persons than rooms (crowding), 14) percentage of households with no vehicle available, and 15) percentage of persons in group quarters. Estimates are created using 2014–2018 (5-year) data from the American Community Survey. The 15 indicators are categorized into four themes: 1) socioeconomic status (indicators 1–4), 2) household composition and disability (indicators 5–8), 3) racial/ethnic minority status and language (indicators 9 and 10), and 4) housing type and transportation (indicators 11–15). Overall SVI includes all 15 indicators as a composite measure.

In the state-level analyses, across overall SVI and all four themes, higher vaccination coverage in high vulnerability counties compared with low vulnerability counties (i.e., equity) was found in two states (Arizona and Montana) (Figure 2) (Supplementary Table, Supplementary Figure 2, https://stacks.cdc.gov/view/cdc/104111). Three other states had higher vaccination coverage in high vulnerability counties than in low vulnerability counties for the overall SVI and three of four themes (Alaska, all except the socioeconomic status theme, and Minnesota and West Virginia, all except the racial/ethnic minority status and language theme). Vaccination disparities were observed in 31 states (overall SVI measure); in 11 of these states, the disparity was found in all four SVI themes.

**FIGURE 2:**
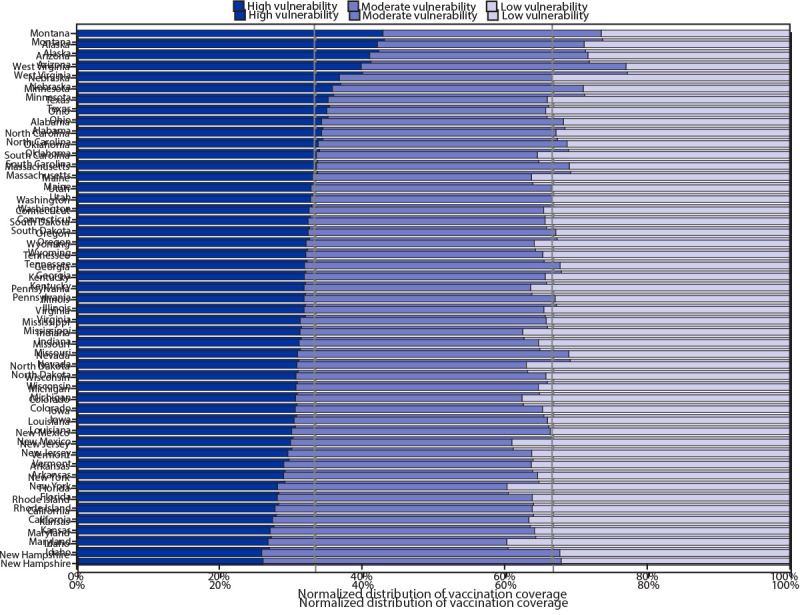
Distribution of county-level* COVID-19 vaccination coverage among persons who received at least one vaccine dose (N = 49,019,117),^†^ by state and overall social vulnerability index (SVI) tertile — United States, December 14, 2020–March 1, 2021 * Counties were assigned to tertiles (low, moderate, and high) for overall SVI. Data are presented as a 100% stacked bar chart (normalized across states), with the length of each bar segment representing the proportion of total vaccination coverage for each SVI tertile. When proportions of vaccination coverage are equal among SVI tertiles, each proportion represents 0.33, represented by the vertical lines. When proportions of vaccination coverage estimates are not equally distributed among SVI tertiles, then proportions do not align with threshold lines representing 0.33. ^†^ Vaccines administered to residents of 48 U.S. states (excluding Delaware, the District of Columbia, and Hawaii) during December 14, 2020–March 1, 2021, and reported to CDC by March 6, 2021.

## Discussion

Ensuring equitable COVID-19 vaccine access is a priority for the U.S. COVID-19 vaccination program.**** In the first 2.5 months of the program, vaccination coverage was lower in high vulnerability counties nationwide, demonstrating that additional efforts are needed to achieve equity in vaccination coverage for those who have been most affected by COVID-19 (*3*). Improving COVID-19 vaccination coverage in communities with high proportions of racial/ethnic minority groups and persons who are economically and socially marginalized is critical because these populations have been disproportionately affected by COVID-19–related morbidity and mortality (*4*–*6*). Monitoring community-level metrics is essential to informing tailored, local vaccine delivery efforts, which might reduce inequities. Public health officials can investigate whether disparities are occurring because of access problems (e.g., vaccine supply, vaccination clinic availability, and lack of prioritization of vulnerable groups) or other challenges, such as vaccine hesitancy. Vaccination promotion, outreach, and administration might focus on high vulnerability populations within counties (e.g., providing resources to federally qualified health centers when socioeconomic disparities are identified).^††††^

Vaccination coverage was consistently lower in high vulnerability counties than in low vulnerability counties for the socioeconomic status indicators (i.e., poverty, unemployment, low income, and no high school diploma); the coverage disparity was largest for the education indicator. However, equal vaccination coverage in counties with low and high social vulnerability was observed for the indicators relating to the percentages of persons who speak English less than well and with persons with a disability, which is encouraging in light of the disproportionate incidence of COVID-19 in these populations.^§§§§^ Higher coverage in counties with large proportions of older adults was consistent with the prioritization of this age group early in the vaccination program; however, the higher coverage in counties with lower percentages of households with a vehicle available was unexpected and warrants further investigation. Despite these positive findings, equity in access to COVID-19 vaccination has not been achieved nationwide.

COVID-19 vaccination equity varied among states. In most states, coverage was higher in low vulnerability counties than in high vulnerability counties. Despite this, states such as Arizona and Montana achieved higher vaccination coverage in high vulnerability counties across SVI metrics. Practices in states with high equity included 1) prioritizing persons in racial/ethnic minority groups during the early stages of the vaccine program implementation, 2) actively monitoring and addressing barriers to vaccination in vulnerable communities, 3) directing vaccines to vulnerable communities, 4) offering free transportation to vaccination sites, and 5) collaborating with community partners, tribal health organizations, and the Indian Health Service.^¶¶¶¶^ More investigation is needed to understand these differences to identify best practices to achieve COVID-19 vaccination equity.

These findings demonstrate that estimates for overall SVI obscured variations among SVI themes and that SVI themes masked variations among indicators within a theme group. In addition, the national coverage estimates by SVI metrics did not capture the wide variation among states. These results highlight the importance of examining individual SVI indicators in addition to the composite SVI measure and themes to monitor equitable vaccine administration. State and local jurisdictions should also consider analyzing SVI metrics at the level of the census tract (when these data are available).

The findings in this report are subject to at least five limitations. First, because specific populations were prioritized for vaccination in each state, the differences observed might be due, in part, to prioritization based on age, occupational exposures, and underlying health conditions. Second, these associations are ecological and reported for population-based metrics rather than individual-level vulnerability data. With only age, sex, and limited race/ethnicity data available at the national level, use of these population-based metrics is an important method to evaluate socioeconomic and demographic disparities. Third, although the geographic unit of analysis was the county, the vulnerabilities and vaccination coverage rates might vary within counties; state and local jurisdictions might prioritize vaccination efforts for high vulnerability communities in smaller geographic units (e.g., census tracts). Fourth, SVI metrics do not include all population characteristics that could be used to identify disparities and focus vaccination efforts, such as lack of Internet access (*7*). Finally, coverage was calculated based on total population, and vaccines authorized for use during the study period were only recommended for persons aged ≥16 or ≥18 years.*****

The results of this study indicate that COVID-19 vaccination coverage was lower in high vulnerability counties than in low vulnerability counties, a finding largely driven by socioeconomic disparities. As vaccine supply increases and administration expands to additional priority groups, CDC, state, and local jurisdictions should continue to monitor vaccination levels by SVI metrics to aid in the development of community efforts to improve vaccination access, outreach, and administration among populations most affected by COVID-19.

**Summary**

**What is already known about this topic?**

COVID-19 has disproportionally affected racial/ethnic minority groups and persons who are economically and socially disadvantaged. Ensuring equitable COVID-19 vaccine coverage is a national priority.

**What is added by this report?**

In the first 2.5 months of the U.S. vaccination program, high social vulnerability counties had lower COVID-19 vaccination coverage than did low social vulnerability counties. Although vaccination coverage estimates by county-level social vulnerability varied widely among states, disparities in vaccination coverage were observed in the majority of states.

**What are the implications for public health practice?**

Continued monitoring of vaccination coverage by social vulnerability metrics is critical for developing tailored, local vaccine administration and outreach efforts to reduce COVID-19 vaccination inequities.

## Footnotes

*
https://www.whitehouse.gov/wp-content/uploads/2021/01/National-Strategy-for-the-COVID-19-Response-and-Pandemic-Preparedness.pdf

†
https://www.cdc.gov/vaccines/covid-19/vaccination-provider-support.html

§ Providers are required to report administration records to the state IIS within 72 hours; 5 additional days of observation were included to account for delays in reporting and transmission of records to CDC.

¶
https://www.census.gov/data/datasets/time-series/demo/popest/2010s-counties-total.html

** https://www.atsdr.cdc.gov/placeandhealth/svi/documentation/SVI_documentation_2018.html; SVI metrics were created using 2014–2018 (5-year) data from the American Community Survey.

†† SVI ranks counties according to 15 social factors (indicators): 1) percentage of persons with incomes below poverty threshold, 2) percentage of civilian population (aged ≥16 years) that is unemployed, 3) per capita income, 4) percentage of persons aged ≥25 years with no high school diploma, 5) percentage of persons aged ≥65 years, 6) percentage of persons aged ≤17 years, 7) percentage of civilian noninstitutionalized population with a disability, 8) percentage of single-parent households with children aged <18 years, 9) percentage of persons who are racial/ethnic minorities (i.e., all persons except those who are non-Hispanic White), 10) percentage of persons aged ≥5 years who speak English “less than well,” 11) percentage of housing in structures with ≥10 units (multiunit housing), 12) percentage of housing structures that are mobile homes, 13) percentage households with more persons than rooms (crowding), 14) percentage of households with no vehicle available, and 15) percentage of persons in group quarters. Estimates were created using 2014–2018 (5-year) data from the American Community Survey. The 15 indicators are categorized into four themes: 1) socioeconomic status (indicators 1–4), 2) household composition and disability (indicators 5–8), 3) racial/ethnic minority status and language (indicators 9 and 10), and 4) housing type and transportation (indicators 11–15). Overall SVI includes all 15 indicators as a composite measure.

§§ Based on data for all counties within the 49 states (excluding Hawaii, which did not systematically report county of residence) included in the national analyses, national SVI metric ranks were created so that each county was ranked against other counties in this sample.

¶¶ State-level SVI ranks excluded jurisdictions with three or fewer counties (Delaware with three counties and DC with one county) and that did not systematically report county of residence (Hawaii). State-level SVI ranks were created for each of the 48 remaining states so that each state’s counties were ranked only among counties in that state; state-level analyses were restricted to overall SVI and the four SVI themes.

*** Each of the 20 SVI metrics (ranks) were divided into tertiles from lowest to highest rank. Counties were classified as follows: 0–0.33: low social vulnerability counties; >0.33–0.66: moderate social vulnerability counties; and >0.66–1: high social vulnerability counties.

††† Among the 52,833,001 persons who received at least one dose of COVID-19 vaccine in the United States, 1.8% (959,301) were excluded, including 1) recipients for whom state of residence was unknown (n = 225,633), 2) residents of eight U.S. territories and freely associated states (n = 475,978) for which SVI data were not available, and 3) residents of Hawaii (257,690).

§§§ Vaccination coverage metrics were normalized so that each tertile’s vaccination coverage was its proportion of total vaccination coverage for that state or national metric.

¶¶¶ 45 C.F.R. part 46, 21 C.F.R. part 56; 42 U.S.C. Sect. 241(d); 5 U.S.C. Sect. 552a; 44 U.S.C. Sect. 3501 et seq.

****
https://www.cdc.gov/vaccines/imz-managers/downloads/Covid-19-Vaccination-Program-Interim_Playbook.pdf

††††
https://www.cdc.gov/vaccines/covid-19/planning/health-center-program.html

§§§§
https://www.cdc.gov/coronavirus/2019-ncov/need-extra-precautions/people-with-disabilities.html

¶¶¶¶ https://dphhs.mt.gov/covid19vaccine; https://www.azdhs.gov/documents/preparedness/epidemiology-disease-control/infectious-disease-epidemiology/novel-coronavirus/vapac-cara-christ-presentation.pdf; https://states.aarp.org/arizona/covid-19-vaccine-distribution; https://www.cnn.com/2021/03/09/us/alaska-covid-19-vaccine-success-trnd/index.html; https://www.usnews.com/news/best-states/articles/2021-03-09/q-a-how-alaska-is-leading-in-covid-19-vaccination-efforts

*****
https://www.cdc.gov/vaccines/covid-19/eua/index.html
